# The Voluntary Hospital: A Retrospect and a Prospect
*Read at the Twelfth Conference of the British Hospitals Association, May 25.


**Published:** 1922-07

**Authors:** W. Thelwall Thomas

**Affiliations:** M.B.E., Ch.M., F.R.C.S., (Senior Surgeon, Royal Infirmary, Liverpool; Professor of Regional Surgery, University of Liverpool)


### THE VOLUNTARY HOSPITAL: A RETROSPECT AND A PROSPECT.*

By W. THELWALL THOMAS, M.B.E., Ch.M., F.R.C.S. (Senior Surgeon, Royal Infirmary, Liverpool; Professor of Regional Surgery, University of Liverpool).

INSTITUTIONS for the treatment of the sick poor have been in existence, in some form or another, so long as records can be traced; in pagan times associated with the temples of the Gods; but their wide distribution in mediæval times was largely due to the Crusades, those world-wide movements of intense religious fervour leading to the foundation of hospitals throughout Europe for the care of the stricken and disabled. The dissemination of leprosy through Western Europe as the result of these hordes of perambulating, nondescript armies and camp followers was of frightful extent, and the erection of leper hospitals (Leprosoria), to deal with the miserable victims covered Europe with institutions for isolation and probably treatment of this scourge. There were over two hundred such in England and Scotland, and at least two thousand in France alone.

The foundation of religious orders devoted to the care of the sick and infirm resulted in the wide diffusion of hospitals throughout the Christian world, but the decay of many such orders as time went on, from lack of funds, led to the slow extinction of many in the fifteenth century. Notable institutions in this country were St. Bartholomew's (1123) and St. Thomas's (1215)—although as early as 794 there is an account of one at St. Albans. These are mentioned by name, although every large religious establishment had its infirmary where medical treatment, suitably interlarded with religious observances and exhortation, were carried out.

The dissolution of the Monasteries in the time of Henry the Eighth put an end at one stroke to these resorts, but as a result of petitions, certain London Hospitals were handed over to the Corporation and City of London by grant. St. Bartholomew's—to mention one—of 100 beds, was endowed with some of its former possessions to the extent of 500 marks per annum, upon condition that the citizens should be bound, yearly for ever, to give the like sum—an intelligent anticipation of the "pound for pound" principle recently enunciated. This was not a voluntary hospital in the modern sense, either in its management or its treatment of chirurgeons, for I find in the quaint phraseology of the times the sentence, "There are also in a kind by themselves, three chirurgeons in the wages of the hospital, giving daily attendance upon the cure of the poor"—who seem to have been fairly well paid, each receiving an annual payment of £20, represented by much more in present value;† some remuneration, now called "an honorarium," continues to the present time. The Corporation of the City retained, and still retains, a considerable share—theoretically, at all events—in its management in virtue of the Lord Mayor and Court of Aldermen and 12 members of the Common Council being ex-officio Governors.

It was nearly two hundred years later that we find the institution of the Voluntary Hospital as we now know it coming into existence, although the Poor Laws of the reign of Queen Elizabeth were passed to deal with the sick poor, but the inefficiency of the poor-house must have been very obvious. In the eighteenth century we find, suddenly and quickly coming into being, the voluntary charities, which have continued ever since—established on a new basis and for a definite object, built by voluntary gifts, maintained by voluntary subscriptions, managed by volunteers, and the treatment undertaken voluntarily by the medical profession.

Between the years 1719 and 1797* there were five hospitals founded in London, 21 in the provincial towns and six in Scotland, and mention must be made of a Sea-bathing Institute for Scrofula in Margate in 1791. In other words, each important provincial town vied with its neighbour in providing a hospital.

It is of interest to inquire into the circumstances of the times to endeavour to ascertain the cause for the marvellous initiation of this wonderfully wide and extensive occurrence. This, I think, will be found in two great simultaneous movements,—one, educational on the part of the medical profession, and the other philanthropic and religious, on the part of the public. The eighteenth century was marked by a great advance in medical learning in this country, Chairs of Anatomy being founded in Edinburgh (1705), Cambridge (1707), Glasgow (1718), and a lectureship in Oxford (1750). Professional Chairs in Medicine were established in Edinburgh (1741) five years after the completion of the Infirmary; in Oxford in 1780, 10 years after its hospital, and a very distinct move forwards followed the inauguration of lectures being delivered in English instead of Latin, in Edinburgh by Cullen in 1757.

Side by side with this educational development we have the great religious revival which spread throughout the land, associated with the names of Dean Berkeley, George Whitefield and the two Wesleys (Charles and John), which awakened the pity of the nation to the terrible condition of the poorer classes, which cannot be better described than by this quota-

* Read at the Twelfth Conference of the British Hospitals Association, May 25.

† For comparison, the cost of diet per patient per day being twopence.

* London: Westminster, 1719; Guy's, 1725; St. George's, 1733; London, 1740; Middlesex, 1745.

Provinces: York, 1710; Salisbury, 1716; Cambridge, 1719; Bristol, 1735; Northampton, 1743; Exeter, 1745; Liverpool, 1745-9; Worcester, 1745; Newcastle, 1751; Manchester, 1753; Chester, 1755; Leeds, 1767; Stafford, 1769; Oxford, 1770; Leicester, 1771; Norwich, 1771; Birmingham, 1778; Sea Bathing Hospital for Scrofula at Margate, 1791.

Scotland: Edinburgh Royal Infirmary, 1729, 1736; Aberdeen, 1739; Dumfries, 1775; Montrose, 1780; Glasgow, 1794, Dundee, 1795.

tion taken from Green's "History of the English People."*

"At the other end of the social scale lay masses of the poor; they were ignorant and brutal to a degree which is hard to conceive, for the increase of population which followed on the growth of towns and the development of commerce had been met by no effort for their religious or educational development. Not a new parish had been created, schools there were none, save the Grammar Schools of Edward VI. and Elizabeth and some newly established 'Circulating Schools' in Wales for religious education. The local peasantry—who were fast being reduced to pauperism by the abuse of the Poor Laws—were left without moral religious training of any sort, and again 'we saw but one Bible in the parish of Cheddar,' said Hannah More, 'and that was used to prop a flowerpot.' Within the towns things were worse. There was no effective police; in great outbreaks the mob of London and Birmingham burnt houses, flung open prisons and sacked and pillaged at their will. The criminal class gathered boldness, and numbers, in the face of ruthless laws, which only testify to the terror of society: laws which made it a capital crime to cut down a cherry tree, and which strung up twenty young thieves of a morning in front of Newgate, while the introduction of gin gave a new impetus to drunkenness. In the streets of London at one time gin-shops invited the passer-by to get drunk for a penny, or dead drunk for twopence."

Describing the soul-stirring effect of the great religious movement, we find:—

"A yet nobler result of the religious revival was the steady attempt, which has never ceased from that day to this, to remedy the guilt, the ignorance, the physical suffering, the social degradation of the profligate and the poor. It was not till the Wesleyan impulse had done its work that the philanthropic impulse began. By her writings, and by her own personal example, Hannah More drew the sympathy of England to the poverty and crime of the agricultural labourer. A passionate impulse of human sympathy with the wronged and afflicted raised hospitals, endowed charities, built churches, sent missionaries to the heathen, supported Burke in his plea for the Hindoo, and Clarkson and Wilberforce in their crusade against the iniquity of the slave trade."

Writing in 1774, Enfield in the second edition of his "History of Leverpool" (*sic*) alludes to the Leverpool Public Infirmary opened in 1749 for the relief of the lame and sick poor. "This establishment was at first formed and has hitherto been conducted on the most liberal principles. It extends its useful charity not only to all proper objects without distinction in the town of Liverpool, but to any whom sickness and misfortune may need to apply for assistance or relief, from whatever quarter they may come, provided only that their cases be such as come within the nature and purpose of the Institution, and that they be recommended by a Trustee: or if they come from a distance, by some person of reputation, but even this in case of sudden accident is not required."

Among the rules this one is found:—"No persons can be admitted who are able to assist themselves and pay for medicines."

The Infirmary began with a flourish, but we soon find the fervid zeal of the benevolent requiring stimulation; by 1767, less than 20 years after the opening, the expenditure exceeded the receipts! The anxieties of Hospital Committees are no new thing, for I find Enfield, evidently inspired by the Treasurer of the day, writing:—

"From the annual reports it appears that the whole stock of the Infirmary amounts to £3,300, the interest of which is only £156 per annum. This, with £20 rent from the Sailors' Hospital, is all the support which this valuable institution has from its own funds. The necessary disbursements upon its present plan—and surely no one would wish to see so useful a design confind to narrower limits—are upwards of £1,100 per annum. It must therefore still receive annual subscriptions and benefactions of the amount of £900, and upwards, to preserve its present state. This plain view of facts may perhaps do more than an eloquent and pathetic harangue to convince the friends of the Institution of the necessity of continuing their contribution, and to induce those who have overlooked this field of charity to add their names to the respectable list of subscribers and benefactors to the Leverpool Infirmary." This has a strangely familiar ring to hospital managers, and might have been written for the last year's annual report—figures alone being altered.

The population of Liverpool was 34,044 in 1770. One shilling per head by the population per annum would have placed the Infirmary in a position of affluence.

The development of the mechanical arts and machinery created a huge manufacturing class, with a large industrial population as workers, and later with the introduction of steam as a motive power, and railways, created a rapid urbanisation of the population, and by the year of the Great Exhibition (1851) one-half of the population of these islands had become aggregated in towns, by 1911 three-fourths. There was no organised attempt to deal with the hospital problem. As the population increased it was left to any one person, or group of individuals, to establish more hospitals as the spirit of humanity, untempered by financial acuity, prompted, and I am sorry to state that the medical man was most frequently the motive force—being nearer the sick and poor person, he allowed his zeal to outrun discretion. Many doctors devoted some hours per week for free consultations and treatment of the poor, either at their own houses or at rooms taken for the purpose. They served the double purpose of helping the poor and extending their own experience; these free clinics, small at their inception when they became uncomfortably large resulted in the calling in the aid of charitably disposed friends to assist; and thus were founded the numerous general and special hospitals met with to-day in all large towns. In Liverpool I have traced the foundation of all hospitals, with the possible exception of the Royal Infirmary, to the members of the medical profession.

In those early days, it is evident from the hospital

* Vol. IV., 1894, p. 1609.

reports that the work of the honorary medical officers occupied only a very small portion of their time per week, a small incident in the week's work, and during my period as house surgeon in 1887 there was only one afternoon per week set apart for operations, and one theatre for surgical work, where the surgical staff, in order of seniority, performed operations on their patients, and it was rarely indeed that the theatre was not empty by 5 o'clock, yet the old hospital had the same number of surgical beds as the present one (150). The total number of surgical operations performed in 1883, with three surgeons and one assistant surgeon on the staff, was only 420—making an average of 105 each for the year ! There was no out-patient department in the modern sense of the term; a few patients occasionally appeared with letters from doctors. The surgeons made one afternoon visit per week lasting two hours, occasionally visiting at odd times on other days for a few minutes if any patient caused special anxiety, or when an emergency or accident appeared (these are included in the number above 420). In 1903 the numbers were 988, and last year a total of nearly 2,000 was reached, yet the staff had only been increased by two assistant surgeons, whose work was largely in the out-patient departments, where large numbers are dealt with.

In recent years the majority of operations are of an importance and magnitude unknown and undreamt of in former years. Confining my observations to the surgical side, the harder worked portion of the hospital, this work has been carried out with the same number of surgical beds (150) as in 1883. During my house-surgency in 1887, under so advanced a surgeon as Sir (then Mr.) Mitchell Banks, the abdominal cavity was not operated upon, and when I mention this you must realise the great advance that has taken place in surgical practice.

How have such large numbers of operations become possible in a hospital with the same number of beds as formerly ? Solely on account of the marvellous strides that have been made in surgery—owing to the developments of antiseptic and aseptic surgery, inaugurated by the late Lord Lister, and advances in the collateral sciences of pathology, bacteriology and medicine. Surgeons have been able by modern technique, *and very much harder work and longer hours*, to bring about what in business parlance is called a greatly increased turnover, and this has for many years kept the need for more beds from becoming acute until comparatively recent years. Each surgeon devotes at least two very long afternoons per week to operations alone, and by utilising what we call here twin theatres has been able by working at high pressure, without any loss of time between operations, to get through a very long list each day.

Parliament has been busy legislating, and incidentally throwing more and more work on the shoulders of the voluntary hospitals for over thirty years, without assisting them in any way, if we except the half-million pounds recently allotted following Lord Cave's Commission, which is serving as a sop to keep them out of the Bankruptcy Court. The Employers' Liability Act, and the Workmen's Compensation Act threw a large volume of work on to the voluntary hospitals. The employment of a workman depended on his being whole or sound, or being made so, if possible, by operation, and all accidents were expected to be treated by the charities. Employers, being business men, contracted with Insurance Companies to undertake their liabilities; which companies, counting on the humanity of the hospitals to treat patients without cost for maintenance or medical treatment, were able to offer low premiums, whereas, were an injured workman to be taken to his own home they would have to consider many items of maintenance and medical and surgical treatment; they were thus relieved from paying funds to hospitals and fees to doctors in estimating bills of costs. Since 1897, the date of the Acts, I have no knowledge of large donations from Insurance Companies out of their high dividends and reserves

The Education Act of 1907 created the salaried School Medical Officer to examine all children in elementary schools for defects and illness, and very efficiently they perform their work, but, unfortunately the powers conferred on education authorities to deal with *treatment* were only *optional*, and the great majority have not availed themselves of these powers—thus throwing more work on the hospitals. (The recent Education Act, in abeyance for the nonce, extends the examinations to secondary schools, and the age to 18.)

Last of all, the National Health Insurance Act of 1911 through a levy on employer, employee and a State contribution confers weekly sickness benefit, treatment for tuberculosis, and maternity benefit plus home or surgery attendance by a panel doctor to more than one-third of the whole population, but contains no provision for nursing, surgery, hospital maintenance, special treatment or medical aid, other than ordinary household attendance, and very minor surgery, thus throwing the really onerous work on the voluntary hospitals for free maintenance and free medical, surgical and special treatment, and it is the steadily increasing pressure of this last weight that is breaking the camel's back. In 1920, with an estimated population of 37,609,600, according to the Ministry of Health Annual Report for that year for England and Wales, there were 13,873,000 insured persons, 36·8 per cent. of the total population, of whom 60 per cent. received medical treatment under the panel system, equal to one-fifth of the population.

Certain conscience-stricken "approved societies" intend giving £250,000 this year—only to pay a small portion of the cost of maintenance of patients in hospital, yet nine of them exclude members resident in Wales ! Taking the number of insured as probably 14,000,000 in 1922, this sum will only represent 4¼d. per insured person per annum !

I quote from THE HOSPITAL AND HEALTH REVIEW for May, p. 226. "To endeavour to draw the remaining majority of the approved societies into this scheme, The British Hospitals Association suggests that a list of members treated in the wards should be forwarded to all approved societies, in the hope that those societies which have not adopted a scheme to benefit the voluntary hospitals may realise the enormous amount of work done for insured persons. As most societies have by now decided upon some method of disposing of their surplus funds, no large success may be expected from such actions."

The large contributions thrown on the employers by the Insurance Act materially militate against expecting large donations to hospitals, some actually imagining the hospitals are being paid, and others frankly asking why hospitals do this work for nothing.

Having been connected with the Royal Infirmary since 1886 and on the honorary surgical staff since 1892, 34 years, the change in the type of patient and the desire for admission are most noticeable. The really "necessitous poor" have very materially diminished, and their place has been taken by the artisan, clerk and small shopkeeper, who can by no means be called "lame and sick poor." The better classes are realising that it is almost impossible to equip private surgical homes on so efficient a scale as a hospital that has been kept up to date with its special departments—to mention only one, the X-ray department, with its costly machinery, frequently replaced owing to rapid advances in that branch of science—and are asking is it right that they are unable to avail themselves of these advantages, for which they are prepared to pay; and I am convinced that the members of the industrial population would rather feel that some financial arrangement were made by some form of definite contribution, which would guarantee payment for services rendered and not expected as a charity. They have a distinct grievance against Parliament, which exacts a weekly contribution from them under the National Health Insurance Act, and yet throws them on to charity in their great need. It may be some time before we approach the American system—but it appears to me that we must travel along their lines soon. There, no person is entitled to occupy a free bed unless, or until, he can prove beyond dispute that he is unable to pay something for the treatment he receives in a hospital ward. They do not consider it the business of the hospital managers to exert themselves to ascertain the circumstances of each patient, because every applicant for relief is bound to present his own credentials, clearly defining to what extent, if at all, he is able to pay for the relief he receives In American ho pitals it will be found that the majority of patients occupy pay beds or paying wards. There being relatively little free medical relief, the American has been educated to feel that no member of the community is entitled to free medical relief, unless or until he becomes so impoverished as to render it altogether impossible for him to pay anything for it—on behalf of himself or any member of his family.

Now, the English practice is the exact contrary; it has become the custom to consider it a part of the duties of the hospital authorities to inquire into the circumstances of the patient (instead of insisting upon each patient providing his credentials). Free relief is now so general in this country that the majority of the population consider it not only not a disgrace, but the most natural thing in the world, when they fall ill to demand and to receive free medical treatment without question or delay. Have we not as committees and doctors been responsible for this—watching our statistics and endeavouring to increase our numbers of patients in order to obtain larger proportions of certain funds?

Burdett, in 1894, wrote, "The medical profession has rendered a great deal too much eleemosynary service in the past," pointing out that in 1890, as the result of a special inquiry, it was found that one-third of the population of Birmingham—apart from the pauper class—altogether received gratuitous medical treatment in the course of each year.

The development of surgical knowledge enables us to do more and more for conditions which only a few years ago were considered beyond our scope, and there is every prospect of further developments. The diffusion of this knowledge to the public creates a steadily increasing demand for surgical aid, and if this had to be rendered gratis in hospital, the amount of time and energy that must be exacted from surgeons will leave only a small amount of time, and a very small percentage of the population willing and able to ask for, and incidentally to remunerate him in order that he may have the wherewithal to obtain sustenance to enable him to live and provide sufficient energy to carry on his gratuitous work.

In so far as we have departed from the original intention of the voluntary hospital we must be prepared to modify the conditions of admission and medical service. The problem is an intricate one, but cannot defy satisfactory solution from the type of hospital committee man it has been my good fortune to be associated with, who has survived the anxieties and worries of his position with urbanity, although much perturbed in spirit. The medical profession can be relied upon to do the just thing, and the public will eventually realise that it is not possible for hospital managers to provide free maintenance for the afflicted for over one-third of the population, nor equitable that the medical profession should have so large a burden of gratuitous service thrown upon it.

